# Oman coral δ^18^O seawater record suggests that Western Indian Ocean upwelling uncouples from the Indian Ocean Dipole during the global-warming hiatus

**DOI:** 10.1038/s41598-018-38429-y

**Published:** 2019-02-13

**Authors:** Takaaki K. Watanabe, Tsuyoshi Watanabe, Atsuko Yamazaki, Miriam Pfeiffer, Michel R. Claereboudt

**Affiliations:** 1 Department of Natural History Sciences, Faculty of Science, Hokkaido University, Sapporo, 060-0810 Japan; 2 KIKAI Institute for Coral Reef Sciences, Kikai town, 891-6151 Japan; 3 Department of Earth and Planetary Sciences, Faculty of Science, Kyusyu University, Fukuoka, 819-0395 Japan; 4 Institut für Geowissenschaften, Christian-Albrechts Universität zu Kiel, 24118 Kiel, Germany; 5 Department of Marine Science and Fisheries, College of Agricultural and Marine Sciences, Sultan Qaboos University, Box 34 Al-Khod 123, Sultanate of Oman

## Abstract

The Indian Ocean Dipole (IOD) is an interannual mode of climate variability in the Indian Ocean that has intensified with 20^th^ century global-warming. However, instrumental data shows a global-warming hiatus between the late-1990s and 2015. It is presently not clear how the global-warming hiatus affects modes of climate variability such as the IOD, and their basin-wide ocean-atmosphere teleconnections. Here, we present a 26-year long, biweekly record of Sr/Ca and δ^18^O from a *Porites* coral drilled in the Gulf of Oman. Sea surface temperature (SST_anom_) is calculated from Sr/Ca ratios, and seawater δ^18^O (δ^18^O_sw-anom_) is estimated by subtracting the temperature component from coral δ^18^O. Our δ^18^O_sw-anom_ record reveals a significant regime shift in 1999, towards lower mean δ^18^O_sw_ values, reflecting intensified upwelling in the western Indian Ocean. Prior to the 1999 regime shift, our SST_anom_ and δ^18^O_sw-anom_ show a clear IOD signature, with higher values in the summer of positive-IOD years due to weakened upwelling. The IOD signature in SST_anom_ and δ^18^O_sw-anom_ disappears with the overall intensification of upwelling after the 1999 regime shift. The inferred increase in upwelling is likely driven by an intensified Walker circulation during the global-warming hiatus. Upwelling in the Western Indian Ocean uncouples from the IOD.

## Introduction

**Figure 1:**
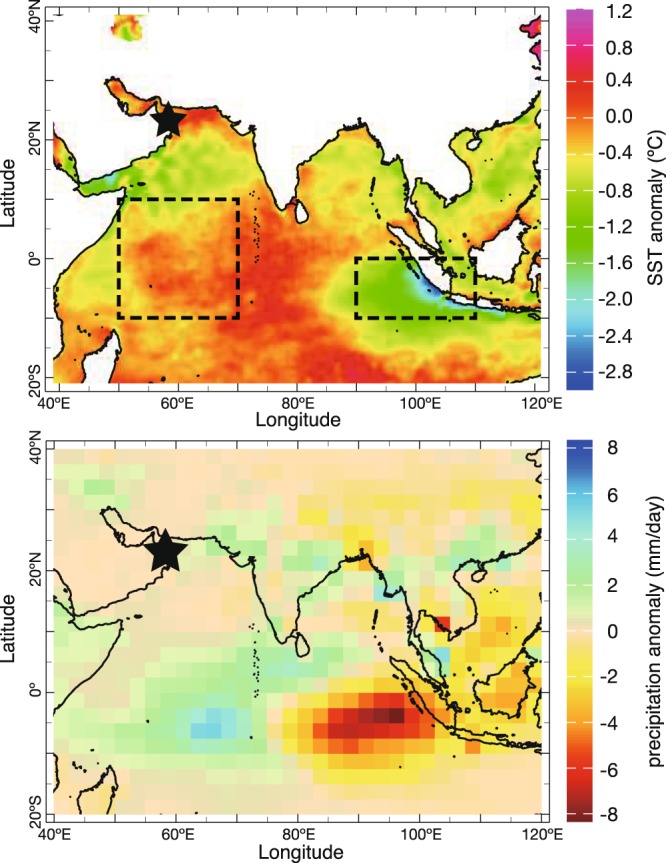
Contour maps show SST and rainfall anomalies in July-November of the IOD event (1994). Boxes mark the eastern and western regions used for calculation the Dipole mode index. Our coral sampling site (star) is shown. SST and precipitation anomalies data were obtained from AVHRR (Advanced Very High Resolution Radiometer^39^) and CAMS_OPI (Climate Anomaly Monitoring System and OLR Precipitation Index^40^), respectively. Contour maps were generated using IRI data library (http://iridl.ldeo.columbia.edu).

The Indian Ocean Dipole (IOD) is an interannual, aperiodic oscillation of sea-surface temperatures in the equatorial Indian Ocean, with positive, neutral and negative phases^1^. The IOD has significant socio-economic impacts in the circum-Indian Ocean region (*e.g*. floods and malaria outbreaks in Kenya^2^; severe droughts in Australia^3^; wildfires in Indonesia and Malaysia^4^). An IOD index has been defined as the difference in sea surface temperature (SST) anomalies between the western Indian Ocean (Arabian Sea) and the eastern Indian Ocean (Sumatra, Indonesia) (Fig. 1)^1^. During the neutral phase of the IOD, SSTs in the western Indian Ocean are colder than in the east, due to intense monsoon-driven upwelling in the Arabian Sea during boreal summer. A positive phase of the IOD is characterized by warmer-than-average SSTs/reduced upwelling in the western Indian Ocean/Arabian Sea, with a corresponding cooling of SSTs/enhanced upwelling in the eastern Indian Ocean. The zonal SST gradient in the Indian Ocean reverses, with warmest temperatures in the west. The negative phase of the IOD brings about opposite conditions and can be seen as an amplification of the neutral phase. The IOD displays a strong asymmetry with the magnitude of the positive IOD being much larger than that of the negative IOD.

Global surface air-temperature observations over the 20^th^ century show a prominent global-warming trend. However, it is not clear how this warming affects modes of ocean-atmospheric interaction such as the IOD. Century-long coral proxy records from Indonesia, the Seychelles and Kenya suggest that the IOD intensified due to a weakened Indo-Pacific Walker circulation following the onset of global-warming during the 20^th^ century^5,6^. This suggests that IOD variability might intensify during future global-warming^5^.

**Figure 2:**
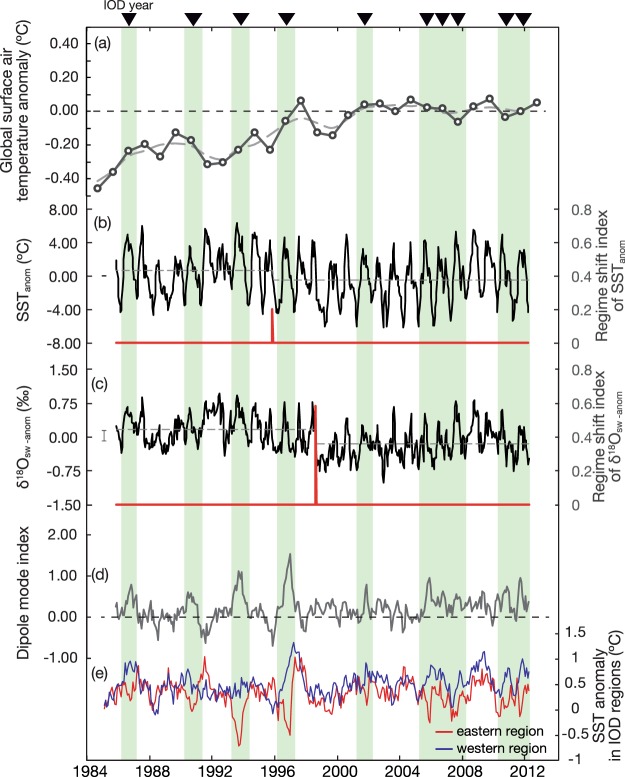
(**a**) Global surface air-temperature anomaly (relative to the period of 1998–2013), the. grey line is a 3-years moving average. Data are from the GISS surface temperature analysis (GISTEMP^41^). Thin dotted line: average global surface air-temperature anomaly during 1998–2013. (**b**) and (**c**) show the biweekly SST_anom_ and δ^18^O_sw-anom_ in the Gulf of Oman (Black line). The dotted lines show the average values during the pre- and post regime shift. The error bars indicate the uncertainties of biweekly SST_anom_ and δ^18^O_sw-anom_. The regime shift index (red line) for both series was generated from sequential *t*-test^37^. (**d**) Dipole mode index. The green patches and triangles show the timing of the IOD years^1^. (**e**) SST anomalies in western and eastern regions of IOD.

However, previous studies have observed that global surface air-temperatures remained relatively constant between the late-1990s and 2015 (Fig. 2a), although climate models predicted continued anthropogenic warming. This so-called global-warming hiatus has received considerable attention^7^. Satellite-based SST data suggest that the main cause of the global-warming hiatus is the Interdecadal Pacific Oscillation (IPO), which is the dominant mode of atmosphere-ocean interactions in the subtropical Pacific. The IPO reversed from a positive to a negative phase in the late 1990s, i.e. the timing of the IPO phase change coincides with the onset of the global-warming hiatus. The negative IPO led to anomalous cooling in the eastern Pacific and this is thought to be a major cause of the global-warming hiatus^7,8^. The regime shift of 1999 has been observed in SST and precipitation data in various regions of the tropics^9,10^. Satellite-based wind stress data suggest that the Indo-Pacific Walker circulation intensified during the global-warming hiatus^11^.

At present, it is poorly understood how the regime shift of the IPO in 1999 and the global-warming hiatus influence the variability and the basin-wide teleconnections of the IOD. As the Walker circulation of the Pacific and Indian Ocean are connected via an “atmospheric bridge” over Indonesia, there is a strong link between climate variations in the Pacific and the Indian Ocean^12^. The IOD primarily reflects a perturbation of the Indian Ocean Walker circulation, but it may be influenced by changes in the Pacific. The coral records previously used to investigate past IOD behavior end in the late 20^th^ century^5^. Hence, they do not encompass the recent global-warming hiatus between 1999 and 2015.

In order to investigate the impact of the global-warming hiatus on the stability of the IOD teleconnection in the western Indian Ocean, we developed biweekly-resolved records (0.5 mm sampling interval) of seawater oxygen isotopes (δ^18^O_sw_) and strontium/calcium ratios (Sr/Ca) from a 26-year long coral core drilled in the Gulf of Oman, Arabian Sea (Fig. 1). Previous coral studies have demonstrated that Sr/Ca reflects SST variations^13,14^, while oxygen isotopes (δ^18^O_coral_) are influenced both by SST and δ^18^O_SW_. Therefore, δ^18^O_SW_ can be estimated by subtracting the SST contribution inferred from Sr/Ca from δ^18^O_coral_. δ^18^O_SW_ mainly reflects the balance between evaporation and precipitation and/or the mixing of water masses with different δ^18^O_SW_ compositions^15^. A previous study of the Oman coral proxy records has demonstrated that they record boreal summer upwelling driven by the Indian/Arabian monsoon from June to September (Indian summer monsoon: ISM)^16^.

## Results and Discussions

### Coral Sr/Ca and δ^18^O_SW_ indicate significant regime shifts in the late 1990s

In the late 1990s, both the SST anomaly (SST_anom_) and the δ^18^O_SW_ anomaly (δ^18^O_SW-anom_) records calculated from the Oman coral show statistically significant regime shifts (Fig. 2b and c). SST_anom_ is calculated directly from measured coral Sr/Ca and δ^18^O_SW-anom_ is estimated by subtracting the Sr/Ca-temperatures from δ^18^O_coral_ (see supplemental information). The sequential *t*-test approach is adopted to identify and to determine the timing and statistical significance of regime shifts (see methods section). The 26-year SST_anom_ record shows a significant regime shift in October 1996 (peak: 0.202; P < 0.01: Fig. 2b). The mean (range) of SST_anom_ is 0.73 ± 2.59 °C (10.96 °C) before 1996 and −0.46 ± 2.71 °C (11.72 °C) after 1996 (Fig. 2b).

The 26-year δ^18^O_SW-anom_ record indicates a major regime shift in July 1999 (peak: 0.583; P < 0.01: Fig. 2c). The mean (range) of δ^18^O_sw-anom_ values is 0.17 ± 0.33‰_VSMOW_ (1.41‰_VSMOW_) before and −0.16 ± 0.34‰_VSMOW_ (1.81‰_VSMOW_) after 1999 (Fig. 2c). The regime shift detected in the δ^18^O_sw-anom_ record in 1999 is more pronounced than that in 1996 in the SST_anom_ record (compare Fig. 2b and c). In addition, SST_anom_ (δ^18^O_SW-anom_) shows a gradual cooling (decrease) over the past-26 years (−0.03 ± 0.01 °C/year and −0.02 ± 0.00‰_VSMOW_/year, respectively).

### δ^18^O_SW-anom_ indicates a major regime shift in 1999 caused by intensified upwelling in the Arabian Sea

The regime shift detected in the δ^18^O_sw-anom_ record (1999) occurs three years later than the regime shift in the SST_anom_ record (1996), and the regime shift in the δ^18^O_sw-anom_ (1999) record is much more pronounced compared to the SST_anom_ record (1996). So what is the correct timing of the regime shift? Which record is more reliable? δ^18^O_sw_ varies depending on the hydrological balance and is closely related to salinity. The regime shift in 1999 observed in δ^18^O_sw-anom_ could be caused by the following two mechanisms: (1) an increase in precipitation relative to evaporation in the region where the coral was sampled and (2) an intensification of upwelling in the western Indian Ocean/Arabian Sea.

To evaluate the potential influence of precipitation on the δ^18^O of sea surface waters^17^, we compare the Omani coral record with *in situ* precipitation rates around the Arabian Sea. The precipitation rates in eastern Oman (Seeb airport: 23.60° N, 58.30° E; GHCN-Month ver. 2: https://www.ncdc.noaa.gov/ghcnm/v2.php) are compared with δ^18^O_sw-anom_. We find that the precipitation rate in Oman decreases after the regime shift in 1999 (average precipitation before the regime shift: 8.5 ± 19.7 mm/month, after the regime shift: 4.7 ± 13.6 mm/month). The observed reduction in precipitation rates would cause more enriched δ^18^O_sw_ values. However, the coral δ^18^O_sw-anom_ record shows a depletion, δ^18^O_sw-anom_ shifts towards lower mean values. This means, the observed regime shift in δ^18^O_sw-anom_ after 1999 is not related to regional precipitation (Oman is an arid area and precipitation is generally very low).

Alternatively, intensified upwelling in the western equatorial Indian Ocean/Arabian Sea may cause the regime shift towards lower mean δ^18^O_sw-anom_ values observed in 1999. Upwelling brings colder water masses with a more enriched-^16^O_sw_ composition to the sea surface^18–20^. In the Arabian Sea, δ^18^O_sw_ decreases with depth (Fig. S3)^18^. The ISM causes strong coastal upwelling along the coast of Somalia and the southern Arabian Peninsula in boreal summer^21^. The upwelled water flows northward, and gyres and eddy systems sweep into the Oman Sea^22^. While upwelling influences both δ^18^O_sw_ and SST, the latter adjusts more quickly to the overlying atmosphere. Hence, the upwelling-related cooling should be weaker, and hence not as distinct in the SST_anom_ record, as the upwelling-related depletion in the δ^18^O_sw-anom_ record. A weaker cooling signature makes it more difficult to accurately determine the timing of the regime shift in the SST_anom_ data. We therefore believe that the Oman δ^18^O_sw-anom_ record is the best indicator of changes in Arabian Sea upwelling, and that an intensification of upwelling occurred in 1999. The year 1999 marks a major regime shift.

### An enhanced Walker Circulation during the global-warming hiatus causes intensified upwelling in the Arabian Sea

The timing of the regime shift in 1999 inferred from the δ^18^O_sw-anom_ record towards colder and lower mean values coincides with the onset of the global-warming hiatus, which lasted from 1999 to 2015. Concurrent shifts in several areas in the late-1990s have been reported in satellite-based SST and precipitation datasets [*e.g*., cooling in the eastern equatorial Pacific^7^; drought in east Africa^9^]. The year 1999 also marks a phase reversal of the IPO, which changed from a positive to a negative polarity.

The intensification of upwelling in the Arabian/Oman Sea following the regime shift in 1999 inferred from our δ^18^O_sw-anom_ record may reflect an intensification of the Walker circulation in the tropical Indo-Pacific^23^. The Walker circulation intensifies during the global-warming hiatus from 1999–2015, caused by low SSTs in the eastern Pacific, a spatial pattern typical of the negative IPO-phase^11^. In contrast, the Walker circulation appears to have weakened during 20^th^ century warming^24^, although this issue is still subject of debate^12^. An intensified Walker circulation as seen during the global-warming hiatus should strengthen upwelling in the Arabian Sea, as the Walker circulation in the Indian and Pacific Ocean is connected by the “atmospheric bridge” over Indonesia. Intensified upwelling in the Arabian Sea leads to cooling and more depleted δ^18^O_sw_ at the sea surface of our coral site.

### Upwelling in the western Indian Ocean uncouples from the Indian Ocean Dipole during the global-warming hiatus

#### The IOD signature in coral proxy data changes following the regime shift in 1999

**Figure 3:**
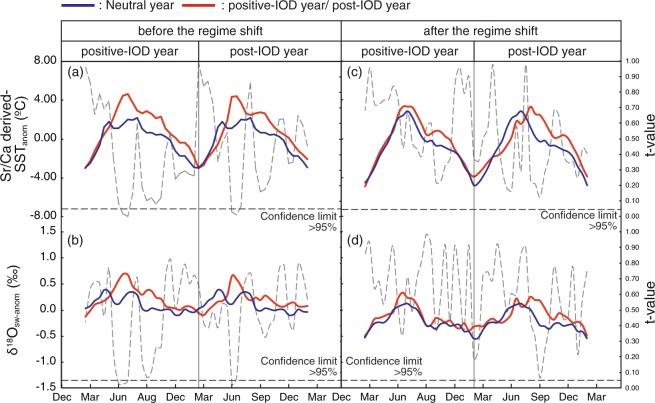
Seasonal SST anomalies (**a**) and δ^18^O_sw_ anomalies (**b**) during neutral years (blue line) and IOD years or post-IOD years (red line) before the regime shift. Dotted line: *t*-test results (neutral years vs. IOD years and neutral years vs. the post-IOD years, respectively). During boreal summer of positive and post IOD years, SST anomalies and δ^18^O_sw_ anomalies are higher than in neutral years. Seasonal SST anomalies (**c**) and δ^18^O_sw_ anomalies (**d**) during neutral years (blue line) and IOD years or post-IOD years (red line) after the regime shift. Dotted line: *t*-test results (neutral years vs. IOD years and neutral years vs. the post-IOD years, respectively). SST anomalies and δ^18^O_sw_ anomalies during neutral, positive-IOD and post IOD years are not significantly different.

In the western Indian Ocean/Arabian Sea positive-IOD events cause reduced upwelling and warm SST anomalies. An intensified Walker circulation as seen during the global-warming hiatus could potentially affect IOD-related upwelling. This can be investigated by comparing of the IOD signatures in the coral proxy data prior to and during the global-warming hiatus. The time series of SST_anom_ and δ^18^O_sw-anom_ are split in two sub-periods (before and after 1999, respectively). The time series are then divided into neutral, positive-IOD and post-IOD years based on the Dipole mode index (Fig. 2d and e)^1^, to capture the biannual variations of SST and winds stress in the Indian Ocean associated with the IOD^25^. Positive-IOD years are defined as years when the Dipole mode index exceeds plus one standard deviation. We then calculate the mean seasonal cycles from the biweekly SST_anom_ and δ^18^O_sw-anom_ values for each of the 3 classes (neutral, positive-IOD and post-IOD) during each sub-period (Fig. 3) and apply a *t*-test to detect statistically significant differences. The Dipole mode index shows 10 positive-IOD events in the 26-year records of SST_anom_ and δ^18^O_sw-anom_ (Fig. 2). Positive-IOD events occur four times prior to the regime shift in 1999, and six times afterwards. Prior to the regime shift in 1999, the mean summer values (June to July) of SST_anom_ and δ^18^O_sw-anom_ are significantly higher during both the positive-IOD year and the post-IOD year (*t*-value < 0.05: Fig. 3a and b). The difference in the mean summer SST_anom_ (δ^18^O_sw-anom_) between neutral and positive-IOD years is 2.79 ± 1.55 °C (0.46 ± 0.32‰_VSMOW_) (Fig. 3a and b). The mean summer values between neutral and post-IOD years differ by 3.07 ± 2.07 °C (SST_anom_) and 0.54 ± 0.37‰_VSMOW_ (δ^18^O_sw-anom_), respectively (Fig. 3a and b). During the global-warming hiatus, which follows the regime shift in 1999, the mean seasonal cycles and the mean summer values of SST_anom_ and δ^18^O_sw-anom_ during positive- and post-IOD years are not significantly different from neutral years (*t*-value > 0.05: Fig. 3c and d), i.e. there is no significant IOD signature in the coral proxy data.

#### The IOD signature in instrumental data changes following the regime shift in 1999

**Figure 4:**
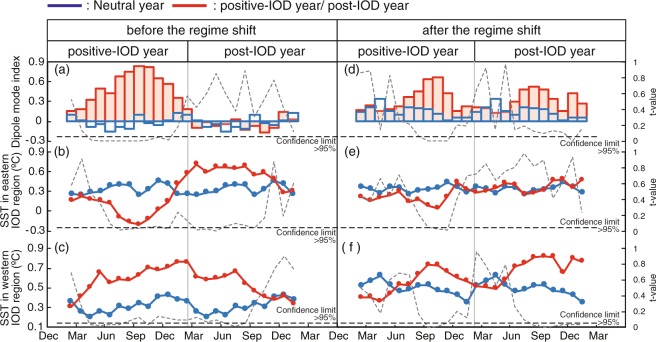
Seasonal Dipole mode index^1^ (**a**) and SST anomalies^1^ in eastern and western regions (**b** and **c**) during neutral years (blue line) and positive-IOD or post-IOD years (red line) before the regime shift. Dotted line: *t*-test results (neutral years vs. positive-IOD years and neutral years vs. the post-IOD years, respectively). Seasonal Dipole mode index (**d**) and SST anomalies in eastern and western  regions (**e** and **f**) during neutral years (blue line) and positive-IOD or post-IOD years (red line) after the regime shift. Dotted line: *t*-test results (neutral years vs. positive-IOD years and neutral years vs. the post-IOD years, respectively).

To further explore the influence of the regime shift in 1999 on the IOD, the Dipole mode index is statistically analyzed using the same methods as for the Omani coral records. Prior to 1999, the Dipole mode index shows significant positive departures during positive-IOD years (*t*-value < 0.05: Fig. 4a), but not during post-IOD years. After 1999, positive departures of the Dipole mode index continue from positive-IOD to post-IOD years (Fig. 4d). SST anomalies in the eastern IOD region (Sumatra, Indonesia) drop during positive-IOD years (due to upwelling of cold water) and increase during post-IOD years (Fig. 4b). The difference between summer SST anomalies of neutral and positive IOD years is 0.57 °C (*t*-value < 0.05) prior to 1999. After 1999, this difference reduces to 0.25 °C (Fig. 4e). In the western IOD region (Arabian Sea) SST anomalies increase during the summer of positive-IOD years and stay warmer than normal until the fall of post-IOD years (Fig. 4c). However, following the regime shift in 1999, the summer SST anomaly differences between neutral and positive-IOD years are much smaller (0.21 °C compared to 0.33 °C prior to 1999). The duration of warm SST anomalies shortens (Fig. 4f).

#### The IOD uncouples from the western Indian Ocean during the global-warming hiatus

Our Omani coral records show that the impact of the IOD on upwelling in the western Indian Ocean/Arabian Sea weakens following the regime shift and the onset of the global-warming hiatus in 1999. The western Indian Ocean/Arabian Sea is sensitive to the IOD, and positive-IODs normally cause warming of surface waters due to reduced upwelling. This is clearly seen in the proxy data prior to 1999, i.e. prior to the onset of the global-warming hiatus: summer SST_anom_ and δ^18^O_sw-anom_ of positive-IOD years are significantly higher than during neutral years. During the global-warming hiatus, however, positive-IOD years are not significantly different from neutral years in the SST_anom_ (δ^18^O_sw-anom_) record. Positive-IOD events are hardly detectable in SST_anom_ and δ^18^O_sw-anom_ after 1999. These results suggest that the IOD weakened during the global-warming hiatus and/or that its impact on upwelling in the western Indian Ocean/Arabian Sea weakened.

**Figure 5:**
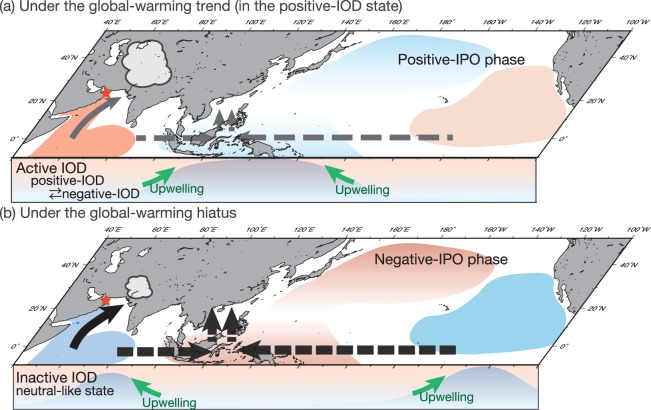
Schematic figures of the climate patterns in the Indian Ocean and the Pacific. The maps were generated using Generic Mapping Tools (GMT ver. 4.5.12^42^). (**a**) In the positive-IOD state during 20th century global-warming and (**b**) in the positive-IOD state during the global-warming hiatus. Map view: color shading indicates the SST gradient (warm: red, cold: blue) in each period. Vertical profiles: color shading indicates the thermocline depth along the equator (warm: red, cold: blue). Black (grey) arrows indicate the stronger (weaker) Walker circulation. Black (grey) solid allows indicate the strong (weak) Indian/Arabian summer monsoon. During the global-warming hiatus, the western Indian Ocean uncouples from the IOD.

The instrumental data support the coral proxy data and suggest that the IOD weakens and uncouples from the western Indian Ocean/Arabian Sea following the onset of the global-warming hiatus. The Dipole mode index^1^ and the SST anomalies from the eastern and western IOD regions^1^ show weaker anomalies during positive-IODs following the regime shift in 1999 (Fig. 4). After 1999, the Dipole mode index indicates weaker but longer-lasting positive IOD events (Fig. 4a and d). Positive SST anomalies continue well into post-IOD years (Fig. 4d). The western Indian Ocean/Arabian Sea shows only weak SST anomalies during positive-IODs. Based on our proxy data we suggest that intensified upwelling in the western Indian Ocean levels out the IOD-driven warming during positive-IOD years, which then appear much more similar to neutral years (Fig. 4f and Fig. 5b). In the eastern Indian Ocean, anomalous cooling (*t*-value < 0.05) during positive-IOD years due to upwelling is also weaker following the regime shift in 1999 (Figs 4e and 5b).

### A stronger Indian summer monsoon strengthens upwelling in the Arabian Sea

The ISM propagates the footprints of the IOD to the western equatorial Indian Ocean and the Arabian Sea (where it is recorded by our coral proxy data)^26^. The Arabian Sea provides the moisture source for ISM summer precipitation in northwestern India. Strong upwelling suppresses evaporation^26^. In turn, however, the primary driver of upwelling in the Arabian Sea is the ISM. A strong ISM intensifies upwelling, enhances cooling (recorded by a decrease in SST_anom_) and brings seawater less depleted in δ^18^O to the sea surface (recorded by a decrease in δ^18^O_sw-anom_)^27^. Note that the latter should be a better indicator of upwelling-related changes, as δ^18^O_sw_ does not adjust as quickly to the overlying atmosphere as SST. To investigate the relationship between the coral proxy data and the ISM, we compare the SST_anom_ (δ^18^O_sw-anom_) record with the monthly maximum precipitation rate in northwestern India (Fig. S4c: precipitation rate data provided from the India Meteorological Department). 3-year moving averages of June to August SST_anom_ and δ^18^O_sw-anom_ (Fig. S4a, b and c) show a positive correlation with the 3-year moving averages of maximum precipitation rate in northwestern India (SST_anom_: r = 0.53, P < 0.01, δ^18^O_sw-anom_: r = 0.71, P < 0.01). These results show that the ISM affects the upwelling intensity in the Arabian Sea during boreal summer.

Prior to the onset of the global-warming hiatus, the ISM responds to SST anomalies in the western Indian Ocean caused by positive-IOD events. Significant differences are observed between neutral and positive-IOD years in the summer values of SST_anom_ (δ^18^O_sw-anom_). These results are consistent with previous work, which suggests that the IOD became stronger during 20^th^ century warming^5,28^. High SST_anom_ and δ^18^O_sw-anom_ values in the summer of the positive-IOD years reflect weak upwelling in the Arabian Sea in response to a weak ISM (Fig. 3a and b). The strength and intensity of the ISM are controlled by the temperature gradient between the Eurasian continent and the Indian Ocean^29,30^. Positive IOD events increase SSTs in the western equatorial Indian Ocean (Fig. 5a) and thereby reduce the temperature gradient between the Eurasia and the Indian Ocean. This weakens the ISM during positive-IOD years.

During the global-warming hiatus, the ISM intensifies and is less sensitive to positive-IODs because the western Indian Ocean uncouples from IOD variability. After 1999, no significant differences are observed in the boreal summer SST_anom_ (δ^18^O_sw-anom_) during neutral and positive-IOD years (Fig. 3c and d), suggesting that the impact of the IOD in the western Indian Ocean/Arabian Sea weakens considerably. Upwelling induced via the ISM seems to have comparable strength in neutral and positive-IOD years (Fig. 5b). Reduced warming in the western Indian Ocean/Arabian Sea during positive-IOD years contributes to the strong ISM observed following the regime shift in 1999. Stronger upwelling reduces SST in the western Indian Ocean/Arabian Sea, and this in turn increases the temperature gradient between the Eurasian continent and the western Indian Ocean during the global-warming hiatus (Fig. S4d and e). This further strengthens the ISM, which in turn causes even stronger upwelling in the Arabian Sea in a positive feedback loop. Our coral proxy data suggests that the basin-wide ocean-atmosphere teleconnections of the IOD are much weaker during the global-warming hiatus, and that the western Indian Ocean/Arabian Sea uncouples from the IOD. This could be a consequence of an enhanced Walker circulation^11^.

In summary, we find evidence for a regime shift in the Gulf of Oman in 1999, which is coincident with a phase change of the IPO and the onset of the global-warming hiatus. The western Indian Ocean/Arabian Sea uncouples from the IOD after the regime shift. We believe this regime shift is caused by an intensified Walker circulation and a stronger ISM during the global-warming hiatus. An uncoupled IOD might also contribute to a slowdown of global-warming during the hiatus period. Upwelling is a mechanism to increase the heat exchange from the ocean to the atmosphere^31^. Upwelling in the western Indian Ocean/Arabian Sea appears to be modulated by decadal IOD fluctuations and should be a subject of further studies to better understand the mechanisms of global-warming.

## Material and Methods

### Oceanographic setting and coral sample

On 23rd, February 2013, we drilled a *Porites* sp. coral colony in the Gulf of Oman (N23°30′, E58°45′: Fig. 1). The coral core is 71 cm long. The Gulf of Oman is located at the outer-rim of the Indian Ocean. Climate and oceanography are strongly influenced by the ISM. In boreal summer, the ISM drives the Somali jet in summer, a strong surface air flow which blows along the Somalian and the southern coast of the Arabian Peninsula. This induces coastal upwelling in the Arabian Sea in every summer^32^. Convective mixing of cold, upwelled water causes strong cooling of surface waters in the boreal summer^21,27,33^.

### Geochemical methods

Detailed methods of the geochemical analysis are described in the supplementary information and in Watanabe *et al*. (2017)^16^. We collected powdered coral samples for geochemical analysis at 0.5 mm intervals along the maximum growth axis of the coral. δ^18^O_coral_ were analyzed with a Finnigan MAT251 stable isotope ratio mass spectrometer system connected with an automated carbonate preparation device (Kiel II) installed at Hokkaido University. Sr/Ca were measured with a SPECTRO CIROS CCD SOP inductively coupled plasma optical emission spectrophotometer installed at Kiel University. δ^18^O_sw_ was calculated from Sr/Ca and δ^18^O_coral_ following Ren *et al*. (2003)^34^. Anomaly records were calculated from Sr/Ca and δ^18^O_sw_ relative to their mean values (SST_anom_ and δ^18^O_sw-anom_). This circumvents the problem of different absolute Sr/Ca-values between corals from different sampling sites^35^. The Sr/Ca ratios were used to develop an age model for all proxies. To obtain a time series with equidistant time steps, the proxy data were interpolated to a biweekly resolution using the AnalySeries software, version 2.0.8^36^.

### Statistical analysis

A sequential *t*-test analysis is applied to the SST_anom_ and δ^18^O_sw-anom_ record. A sequential *t*-test analysis can automatically detect multi occurrences of regime shifts and is less sensitive to the presence of trends^37^. In sequential *t*-test analysis, the timing of regime shifts is identified with a Student’s *t*-test. The “cut-off length” determines the length of detected regime shifts^37^. A longer cut-off length identifies few events with major signals (conversely, a shorter cut-off length identifies many small events)^37,38^. In this study, we chose a long cut-off length (13 years) to identify the year of major change in the SST_anom_ and δ^18^O_sw-anom_ records. The timing of regime shifts is detected at the 1% probability level. To evaluate the magnitude of the regime shifts, regime shift indexes for SST_anom_ and δ^18^O_sw-anom_ are used^37^.

## Supplementary information

Supplemental information
