# Supplementary material for: Oman coral δ18O seawater record suggests that Western Indian Ocean upwelling uncouples from the Indian Ocean Dipole during the global-warming hiatus
Source: Sci Rep. 2019 Feb 13;9:1887. doi: 10.1038/s41598-018-38429-y (PMC6374511; doi:10.1038/s41598-018-38429-y)
Supplement: Supplementary file 1 — Supplemental information [file 41598_2018_38429_MOESM1_ESM.docx]

Supporting Information for

Oman coral δ^18^O seawater record suggests that Western Indian Ocean upwelling uncouples from the Indian Ocean Dipole during the global-warming hiatus

Takaaki K. Watanabe^1^, Tsuyoshi Watanabe^1,2^*, Atsuko Yamazaki^1,2,3^, Miriam Pfeiffer^4^, Michel R. Claereboudt^5^

^1^ Department of Natural History Sciences, Faculty of Science, Hokkaido University, Sapporo, 060-0810, Japan.

^2^ KIKAI Institute for Coral Reef Sciences, Kikai town, 891-6151,

^3^ Department of Earth and Planetary Sciences, Faculty of Science, Kyusyu University, Fukuoka, 813-8581, Japan.

^4^ RWTH Aachen, Wuellnerstrasse2, 52056, Aachen, Germany.

^5^ Sultan Qaboos University, College of Agricultural and Marine Sciences, Department of Marine Science and Fisheries, Box 34, Al-Khod, 123, Sultanate of Oman.

**Contents of this file**

Text S1

Figures S1 to S4

Tables S1 to S3

**Introduction**

The supplementary information includes one text, four figures (Fig. S1, Fig. S2, Fig. S3 and Fig. S4) and three tables (TableS1, Table S2 and Table S3). We describe the detailed methods for the geochemical analysis and statistical analysis, and the geochemical analytical results in the text and Fig. S1. We show and discuss the temperature dependency of Sr/Ca and oxygen isotopes in the Omani coral using sea surface temperature in Fig.S2, TableS1, TableS2 and Table S3. We show the depth profile of oxygen isotope in seawater from the Arabian Sea on Fig. S3. Coral and satellite records related to the Indian/Arabian monsoon in summer are shown on Fig.S4

Text S1.

**1. Methods**

**1-1. Geochemical methods**

**1-1-1 Sample preparation**

A *Porites sp*. coral colony was drilled in the Gulf of Oman (23°30’ N, 58°45’ E) on February 23, 2013. The Porites colony was living at a 2m water depth in a small bay (Bandar Khayran). In total, the length of the coral core was 71 cm. The coral core was sliced into 5-mm-thick slabs. We took X-radiographs of the coral slabs to identify the maximum growth axis of the coral. We prepared a ledge of 1.5 mm thickness along the maximum growth axis. Coral powder was obtained along the ledge in 0.5 mm intervals.

**1-1-2 Oxygen isotope measurement**

100 μg (± 20μg) of coral powders were weighed for oxygen stable isotope analysis. The sample powder was reacted with 100% H_3_PO_4_ at 70ºC in an automated carbonate device (Kiel II). The δ^18^O_coral_ was analysed with Finnigan MAT251 stable isotope ratio mass spectrometer installed at Hokkaido University. The analytical precision for δ^18^O_coral_ were 0.07‰ based on replicate measurements of the NBS-19 (1σ, n = 40).

**1-1-3 Sr/Ca ratio measurement**

Approximately 250μg of coral powder was taken for the Strontium/Calcium ratio (Sr/Ca) measurements. The weighed coral powder was dissolved in 4 mL of HNO3. The sample solution for the Sr/Ca measurement was prepared via serial dilution with 2% HNO3 to get a Ca concentration of 8 ppm. The Sr/Ca were measured with a SPECTRO CIROS CCD SOP inductively coupled plasma optical emission spectrophotometer installed at Kiel University. Analytical precision of the Sr/Ca determinations was 0.07% RSD or 0.01 mmol×mol^-1^ (1σ).

**1-1-4 Data analysis**

The Sr/Ca and Advanced Very High-Resolution Radiometer (AVHRR) satellite-SST dataset^1^ were used for developing an age model for coral proxy. Seasonal minima and maxima of Sr/Ca were tied to the maxima and minima of SST, respectively. The Sr/Ca-SST thermometer was established using tie-points of Sr/Ca and SST. δ^18^O_SW_ was estimated by subtracting the temperature component (calculated from Sr/Ca) from δ^18^O_coral_ following the method proposed by Ren *et al*. (2003). To obtain a time series with equidistant time steps, the proxy data were interpolated to a biweekly resolution using the AnalySeries software, version 2.0.8^2^.

**1-2 Statistical method**

To detect the regime shift, and to evaluate its timing and statistical significance, we adopted the sequential t-test approach^3^. For time-series of SST_anom_ and δ^18^O_sw-anom_, [X*k*, k = 1, 2, …, n], the mean of the first regime (R1), X_R1_ is determined as

$${\bar{\text{X}}}_{\text{R1}}\text{= }\sum_{\text{k}\text{=1}}^{\text{l}} \text{X}\text{k}\text{ }\left( \text{1≤k≤}\text{l} \right)$$

where *l* is the “cut-off length” of regimes to be determined the mean. The cut-off length controls the time-scale of the detected regime shift. The cut-off length determines the minimum length of the regime shift not altering the magnitude of the shift^3^. To identify the major change of regime in all time-series, we chose a long cut-off length (13 years). The difference from the mean of next regimes (*R2*) that would be statistically significant (Student’s *t*-test) is given by

$$\text{diff=}\text{t}\sqrt{\text{2}\text{σ}_{\text{l}}^{\text{2}}\text{/}\text{l}}$$

where *t* is the value of the *t*-distribution with 2*l* – 2 degrees of freedom at 1 % probability level. 2σ^2^*_l_* is the average variance for the intervals during the cut-off length. Every X*k* (*k* ≥ *l* + 1) is evaluated in a sequential order. If X*k* is within the range of ±diff (from X_R-1_ - diff to X_R-1_ + diff), the X_R-1_ is recalculated with the X*k* value and *l*-1 previous X*k* values. If X*k* is out of the range of ±diff, it is considered as a possible starting point (*k = j*) of the next regime (*R2*). The regime shift index (RSI) is determined to confirm the significance of the next regime stating point at year *j*,

$$\text{RSI}_{\text{i,j}}\text{=}\sum_{\text{i}\text{=}\text{j}}^{\text{j}\text{+}\text{m}} \begin{aligned} \frac{\text{x}_{\text{i}}\text{-}\left( \text{X}_{\text{R1}}\text{+diff} \right)}{\text{l}\text{σ}_{\text{l}}}\text{ } \\ \text{ } \end{aligned}$$

(m = 0,1…, *l*-1)

If the RSI has the same signal at year *j* with evaluation using “diff”, it increases the confidence of the shift occurrence. The evaluation for the next regime shift was conducted from the following data of the current regime until all the time-series were evaluated.

**2. Results**

We determined Sr/Ca and δ^18^O_coral_ from 664 powder samples. Sr/Ca and δ^18^O_coral_ showed 26 distinct annual cycles. The average of Sr/Ca was 9.28 (mmol×mol^-1^). The maxima and minima of Sr/Ca values were 9.56 and 8.98 (mmol×mol^-1^). The δ^18^O_coral_ averaged -4.33 (‰_VPDB_) and ranged from -4.92 to 3.41 (‰_VPDB_).

The regression line of AVHRR-SST and Sr/Ca was established using seasonal maxima and minima, as follows:

Sr/Ca (mmol×mol^-1^) = -0.044 ± 0.003 SST (ºC) + 10.46 ± 0.18 (r = 0.95: P < 0.01)

The regression line of AVHRR-SST and δ^18^O_coral_ was established using seasonal maxima and minima assuming δ^18^O_coral_ reflect only SST, as follows:

δ^18^O_coral_ (‰_VPDB_) = - 0.104 ± 0.005 SST (ºC) – 1.28 ± 0.14 (r = -0.92 P < 0.01)

Satellite based sea surface temperature (SST) in the Gulf of Oman did not reflect low SST excursions in summer measured by CTDs^4^. In order to evaluate effect of uncertainty in satellite-based SST, we established SST dependency of Sr/Ca and δ^18^O_coral_ using seasonal minima (maxima) (TableS1). The slope of SST-Sr/Ca (δ^18^O_coral_) regression using both winter and summer peaks was similar with the slope using winter peaks, while SST-Sr/Ca (δ^18^O_coral_) regression using summer peaks could not established (non-significant).

Our SST-Sr/Ca regression using both winter and summer peaks would be suitable for estimation of SST-variations, because our slope value of Sr/Ca-SST regression was consistent within the range of the previous published values (from -0.08 to -0.04 mmol×mol^-1^) (Fig.S2-a and TableS2). Our slope value of δ^18^O_coral_ -SST regression was higher than the previously reported values (Fig.S2-b and TableS3). Only Timor coral δ^18^O showed similar slope values, and this record was shown to record seasonal-scale δ^18^O_SW_/salinity variations^5^. At Timor, maximum SST coincided with maximum salinity, so the seasonal cycle of coral δ^18^O with respect to temperatures was dampened^6^. The coral δ^18^O-temperature relationship of the Omani coral suggested that δ^18^O_SW_ affects δ^18^O_coral_ in either winter or summer, in the sense that colder (warmer) SSTs coincided with depleted (enriched) δ^18^O_SW_.

We calculated SST anomaly (SSTanom) using the slope of Sr/Ca-SST (-0.044 ± 0.003 mmol×mol^-1^/ºC : Watanabe *et al*., 2017) and anomalies of Sr/Ca relative to average Sr/Ca (9.28 mmol×mol^-1^). The uncertainty of biweekly SST_anom_ estimates was 0.03ºC, which was calculated based by combining the analytical error of the Sr/Ca ratios (± 0.01 mmol×mol^-1^) and the slope error (± 0.003 mmol×mol^-1^/ºC). We calculated anomalies of δ^18^O_sw_ (δ^18^O_sw-anom_) by subtracting the 26 year-average δ^18^O_SW_ (1.12‰_VSMOW_) from δ^18^O_SW_. The uncertainties of biweekly δ^18^O_sw-anom_ were 0.12 ‰_VSMOW_ following^7^.

**Reference list**

1. Reynolds, R. W. *et al.* Daily high-resolution-blended analyses for sea surface temperature. *Journal of Climate* **20,** 5473–5496 (2007).
2. Paillard, D., Labeyrie, L. & Yiou, P. Macintosh Program performs time-series analysis. *Eos, Transactions American Geophysical Union* **77,** 379 (1996).
3. Rodionov, S. N. A sequential algorithm for testing climate regime shifts. *Geophysical Research Letters* **31,** n/a-n/a (2004).
4. Watanabe, T. K., Watanabe, T., Yamazaki, A., Pfeiffer, M. & Claereboudt, M. R. Past summer upwelling events in the Gulf of Oman derived from a coral geochemical record. 1–7 (2017). doi:10.1038/s41598-017-04865-5
5. Cahyarini, S. Y., Pfeiffer, M., and Dullo, W. Improving SST reconstructions from coral Sr / Ca records : multiple corals from Tahiti ( French Polynesia ). 31–40 (2009). doi:10.1007/s00531-008-0323-2
6. Cahyarini, S. Y., Pfeiffer, M., Timm, O., Dullo, W. & Schönberg, D. G. Reconstructing seawater d 18 O from paired coral d 18 O and Sr / Ca ratios : Methods , error analysis and problems , with examples from Tahiti ( French Polynesia ) and Timor ( Indonesia ). **72,** 2841–2853 (2008).
7. Nurhati, I. S., Cobb, K. M. & Di Lorenzo, E. Decadal-scale SST and salinity variations in the central tropical pacific: Signatures of natural and anthropogenic climate change. *Journal of Climate* **24,** 3294–3308 (2011).
8. Gagan, M. K. *et al.* Temperature and Surface-Ocean Water Balance of the Mid-Holocene Tropical Western Pacific. *Science* **279,** 1014–1018 (1998).
9. Marshall, J. F. & McCulloch, M. T. An assessment of the Sr/Ca ratio in shallow water hermatypic corals as a proxy for sea surface temperature. *Geochimica et Cosmochimica Acta* **66,** 3263–3280 (2002).
10. Zinke, J., Dullo, W., Heiss, G. A. & Eisenhauer, A. ENSO and Indian Ocean subtropical dipole variability is recorded in a coral record off southwest Madagascar for the period 1659 to 1995. **228,** 177–194 (2004).
11. Felis, T. *et al.* Increased seasonality in Middle East temperatures during the last interglacial period. *Nature* **429,** 164–168 (2004).
12. Sagar, N. *et al.* High-resolution Sr/Ca ratios in a Porites lutea coral from Lakshadweep Archipelago, southeast Arabian Sea: An example from a region experiencing steady rise in the reef temperature. *Journal of Geophysical Research: Oceans* **121,** 252–266 (2016).
13. Quinn, T. M., Taylor, F. W., Crowley, T. J. & Link, S. M. Evaluation of sampling resolution in coral stable isotope records: A case study using records from New Caledonia and Tarawa. *Paleoceanography* **11,** 529–542 (1996).
14. Felis, T. *et al.* A coral oxygen isotope record from the northern Red Sea documenting NAO, ENSO, and North Pacific teleconnections on Middle East climate variability since the year 1750. *Paleoceanography* **15,** 679–694 (2000).
15. Pfeiffer, M., Timm, O., Dullo, W. C. & Podlech, S. Oceanic forcing of interannual and multidecadal climate variability in the southwestern Indian Ocean: Evidence from a 160 year coral isotopic record (La Reunion, 55ºE, 21ºS). *Paleoceanography* **19,** 1–14 (2004).
16. Wessel, P. & Smith,W. H. F. Free Software Helps Map and Display Data. *Eos Trans. AGU.* **72**(41), 441–446 (1991).
17. Schmidt, G. a. Forward Modeling of Carbonate Proxy Data from Planktonic Foraminifera using Oxygen Isotope Tracers in a Global Ocean Model. **14,** 482–497 (1999).
18. Janowiak, J. E., & Xie, P. CAMS–OPI: A global satellite–rain gauge merged product for real-time precipitation monitoring applications. Journal of Climate, **12**(11), 3335-3342 (1999).

**Table S1**

The SST dependency of the Sr/Ca and δ^18^O_coral_ in coral skeletons using summer, winter peaks or both peaks. The Sr/Ca (δ^18^O_coral_) - SST regression line applied for this study is showed with bold letter.

**Table S2**

Temperature dependency of the Sr/Ca ratio in coral skeletons.

**Table S3**

Temperature dependency of δ^18^O in coral skeletons.

**Figure S1**


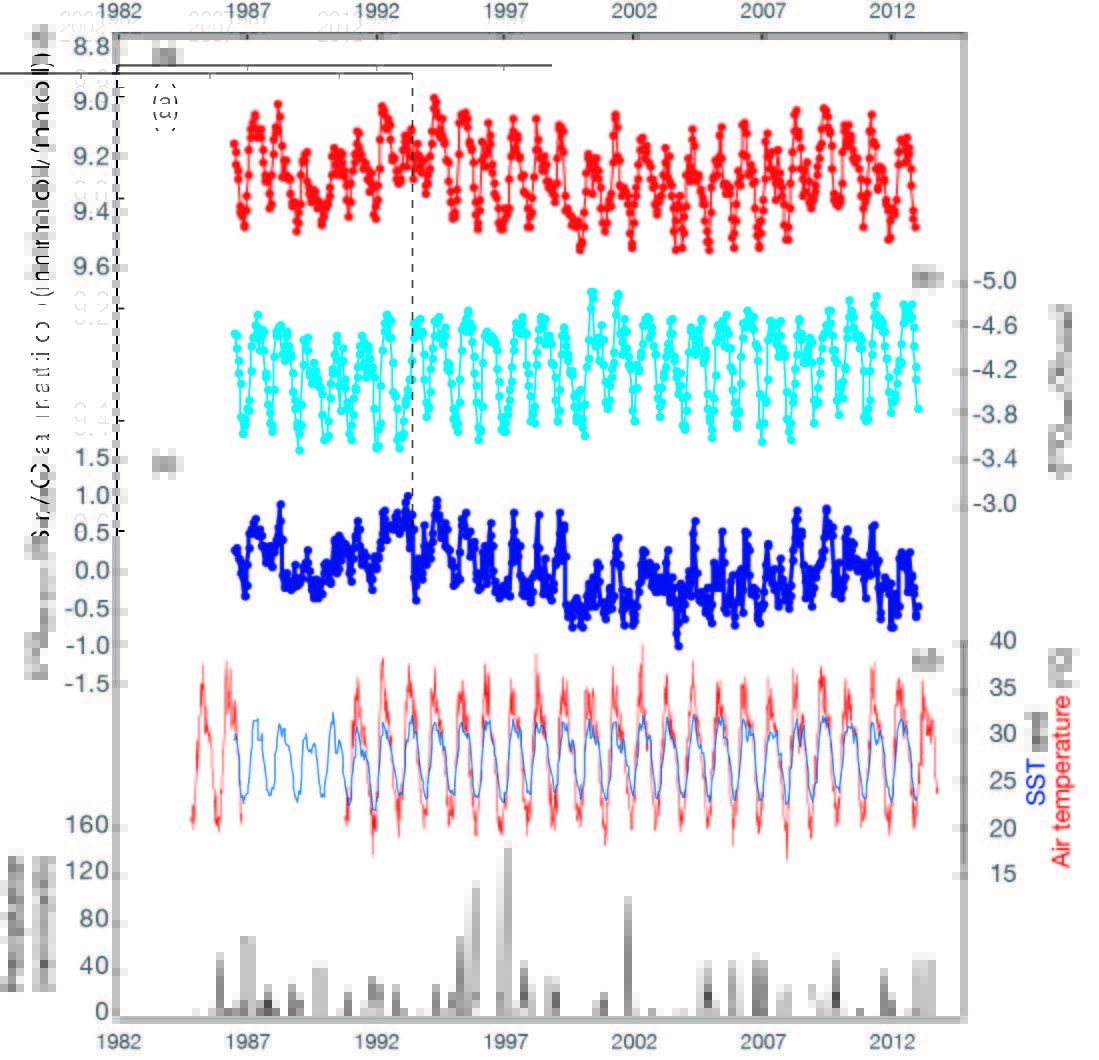


Biweekly records of the Sr/Ca ratios (a), δ^18^O_coral_ (b) and δ^18^O_SW-anom_ (c), AVHRR-SST in the Gulf of Oman (blue line)^1^ and observational air-temperature at Seeb airport (red line: dataset from World Meteological Organization station: https://www7.ncdc.noaa.gov/CDO/cdo ) (d) and observational precipitation at Seeb airport (GHCN-Monthly ver. 2^18^).

Figure S2

Calibration of the Sr/Ca ratio and δ^18^O_coral_ thermometer (a and b). Circle-dots indicate the seasonal maxima and minima of the Sr/Ca (δ^18^O_coral_), which are tied with the maxima and minima of SST, respectively (purple dots: minima of SST; green dots: maxima of SST). Cross-dots show the data points between all data points of Sr/Ca ratio (δ^18^O_coral_).

(a) The red line shows our Sr/Ca-SST calibration line using both summer and winter peaks record in the *Porites* coral from the Gulf of Oman. The purple line (green dotted line) indicates our Sr/Ca-SST calibration line using summer (winter) peaks recorded in the Porites coral from the Gulf of Oman. The gray lines indicate the previous published Sr/Ca-SST calibration lines (TableS1).

(b) The blue line shows our δ^18^O_coral_-SST calibration line using both summer and winter peaks recorded in the *Porites* coral from the Gulf of Oman. The purple line (green dotted line) indicates our δ^18^O_coral_-SST calibration line using summer (winter) peaks recorded in the Porites coral from the Gulf of Oman. The gray lines indicate the previous published δ^18^O_coral_-SST calibration line (TableS2). The slope of δ^18^O_coral_-SST calibration line using both summer and winter peaks in our Omani coral is similar to the Timor δ^18^O_coral_ -SST slope.

**Figure S3**

Depth profile of δ^18^O_sw_ data from the Arabian Sea (a). Data from Schmidt *et al*., 1999^16^ (https://data.giss.nasa.gov/cgi-bin/o18data/geto18.cgi). The circle dots on the map indicate water sampling sites (b). The star dot shows our coral sample site. The maps were generated using Generic Mapping Tools (GMT ver. 4.5.12^17^)

**Figure S4**


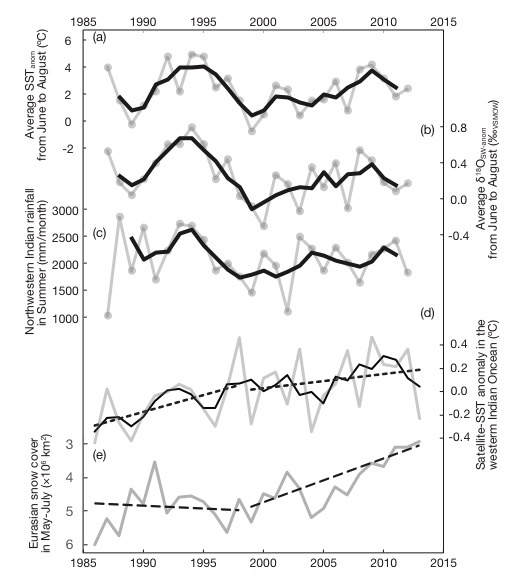


Coral, satellite and in situ records related to the SWM (gray line: seasonal average values, thick black line: 3-year moving averages and dotted line: trend line estimated by linear regression before and after the regime shift in 1999).

(a) Average SST_anom_ from June to August. (b) Average δ^18^O_sw-anom_ from June to August. (c) Maximum rainfall from June to August in northwestern Indian. (d) Satellite derived SST anomaly in the late-spring (May-June) in the western equatorial Indian Ocean (5ºS-5ºN, 55-65Eº). (e) The Eurasian snow cover in late-spring (May-June).

SST anomalies from the western Indian Ocean and Eurasian snow cover suggested the temperature gradient between the Eurasian and Indian Ocean increase.
